# TREM1/3 Deficiency Impairs Tissue Repair After Acute Kidney Injury and Mitochondrial Metabolic Flexibility in Tubular Epithelial Cells

**DOI:** 10.3389/fimmu.2019.01469

**Published:** 2019-07-09

**Authors:** Alessandra Tammaro, Angelique M. L. Scantlebery, Elena Rampanelli, Cristiana Borrelli, Nike Claessen, Loes M. Butter, Alessandra Soriani, Marco Colonna, Jaklien C. Leemans, Mark C. Dessing, Sandrine Florquin

**Affiliations:** ^1^Amsterdam UMC, University of Amsterdam, Amsterdam, Netherlands; ^2^Laboratory Affiliated With Istituto Pasteur Italia - Fondazione Cenci Bolognetti, Department of Molecular Medicine, Sapienza University of Rome, Rome, Italy; ^3^Center for Life Nano Science, Istituto Italiano di Tecnologia, Rome, Italy; ^4^Department of Pathology and Immunology, Washington University School of Medicine, St. Louis, MI, United States

**Keywords:** maladaptive repair, mitochondrial metabolism, tubular cell senescence, epithelial innate immunity, renal repair, ischemia/reperfusion injury

## Abstract

Long-term sequelae of acute kidney injury (AKI) are associated with incomplete recovery of renal function and the development of chronic kidney disease (CKD), which can be mediated by aberrant innate immune activation, mitochondrial pathology, and accumulation of senescent tubular epithelial cells (TECs). Herein, we show that the innate immune receptor Triggering receptor expressed on myeloid cells-1 (TREM-1) links mitochondrial metabolism to tubular epithelial senescence. TREM-1 is expressed by inflammatory and epithelial cells, both players in renal repair after ischemia/reperfusion (IR)-induced AKI. Hence, we subjected WT and TREM1/3 KO mice to different models of renal IR. TREM1/3 KO mice displayed no major differences during the acute phase of injury, but increased mortality was observed in the recovery phase. This detrimental effect was associated with maladaptive repair, characterized by persistent tubular damage, inflammation, fibrosis, and TEC senescence. *In vitro*, we observed an altered mitochondrial homeostasis and cellular metabolism in TREM1/3 KO primary TECs. This was associated with G2/M arrest and increased ROS accumulation. Further exposure of cells to ROS-generating triggers drove the cells into a stress-induced senescent state, resulting in decreased wound healing capacity. Treatment with a mitochondria anti-oxidant partly prevented the senescent phenotype, suggesting a role for mitochondria herein. In summary, we have unraveled a novel (metabolic) mechanism by which TREM1/3 deficiency drives senescence in TECs. This involves redox imbalance, mitochondrial dysfunction and a decline in cellular metabolic activities. These finding suggest a novel role for TREM-1 in maintaining tubular homeostasis through regulation of mitochondrial metabolic flexibility.

## Introduction

Acute kidney injury (AKI) remains a financial burden for society due to its high morbidity and mortality rates. Recent epidemiological and clinical studies have linked AKI to an increased risk of developing chronic kidney disease (CKD) (1). The ischemic damage experienced during transplant procedures is a common cause of AKI. The proximal tubular epithelial cells (TECs), have a high metabolic rate, thus, sensitive to ischemic injuries (2). As such, being metabolically flexible provides an important advantage to quickly respond to environmental changes and ensure a healthy tubular function. Indeed, the renal epithelium has the ability to regenerate after injury; surviving TECs enter the cell cycle within a few hours following AKI, in order to promote tubular proliferation and return to homeostasis (3–5). This is the so-called “adaptive repair” post-AKI, which results in the recovery of renal function. However, maladaptive repair might also occur, as a consequence of cell cycle arrest or senescence in TECs often accompanied by an increased secretory phenotype, which contributes to persistent inflammation, loss of renal function and renal fibrosis (6, 7). Cellular senescence is an irreversible growth arrest which can be triggered by DNA damage, oxidative stress, or mitochondrial injury. TECs are densely packed with mitochondria because of their reabsorptive functions, which require high energy expenditure. Thus, it is not surprising that mitochondrial dysfunction in TECs is thought to be a pathogenic mechanism underlying the AKI-CKD transition (8–10).

Senescence tubular cells accumulate in diseased and aged kidney (11). Additionally, in responses to danger signals released during IR-induced AKI, TEC-associated pattern recognition receptors (PRRs) function as sentinels to dictate epithelial inflammation and repair, a regulatory process which might also be affected during aging.

Triggering Receptor Expressed on Myeloid cells-1 (TREM-1) is an amplifier of the inflammatory response elicited by TLR4, and we have previously published that following renal IR, TREM-1 is upregulated in the early phase of damage, due to the infiltration of granulocytes, expressing high levels of TREM-1 (12, 13). Similar to the role of TLR4 in renal IR, damage induced by inflammatory cell-associated TREM-1 plays a dispensable role in renal IR (12). During sterile inflammation, TLR4 and TREM-1 expression is also detectable on epithelial cells (13, 14). Epithelial TLR4-mediated inflammation appears detrimental during the acute phase of injury after renal IR but essential for tubular regeneration (15, 16). Besides its role in immune regulation, TREM-1 has also been linked to cell survival and mitochondrial integrity (17). Given these evidences, we hypothesized that TREM-1 could play a role in renal repair after AKI. Therefore, we subjected WT and TREM1/3 double KO animals to renal IR injury and investigated the tissue repair mechanisms herein. The TREM1/3 KO mice were chosen as they represent a better model for translational studies as *Trem3* appears to be a pseudogene in humans.

The present study reports a novel mechanism through which the immune receptor TREM1/3 maintains tubular integrity by suppressing senescence programs herein, limiting the maladaptive repair following IR. TREM1/3 provides TECs with a metabolic flexibility necessary for proliferation, which favors renal regeneration after IR.

## Materials and Methods

### Reagents and Antibodies

Rat anti-mouse F4/80 (BD pharmigen), mouse anti-mouse αSMA (clone 1A4, DAKO), rabbit-polyclonal collagen type 1 (Genetex), rabbit anti-Ki67 (Sp6, Neomarkers), rabbit anti-cleaved caspase-3 (Cell signaling), goat polyclonal-anti TREM-1 (Santa Cruz), rabbit polyclonal-anti TREM-1 (Abcam), mouse anti-phosphorylated histone H3 (Phospho S10) (Abcam), anti-Tom20 (Clone 2Fb.1) (Millipore), anti-p21/WAF/Cip1 (Millipore), rabbit anti-biotinylated TREM-1 antibody (50D1 clone; gift from Colonna Lab), rabbit anti-Mitofusin2 (D2D10) (Cell signaling), rabbit anti-AMPKα and rabbit anti-phospho-AMPKα (Cell signaling), rabbit anti-mouse PKM2 (Cell signaling), anti- mouse Alexa fluor488 (Invitrogen), donkey-anti-rabbit Alexa 594 (Jackson), hoechst 33342 (Sigma), streptavidine-APC (BD), mouse KC, MCP-1, sTREM-1 duo-set Elisa (R&D), streptavidin-HRP (Sanquin), TMB (Sigma), collagenase from *clostridium histolyticum*, type 1A (Sigma), propidium Iodide (Molecular probes), senescence-associated β-galactosidase (SA-β-Gal) staining kit (cell signaling), C_12_FDG (5-Dodecanoylaminofluorescein Di-β-D-Galactopyranoside) (Invitrogen), fixable viability stain 780 (BD), bafilomycin A1 from *Streptomyces griseus* (Sigma), MITO-ID membrane potential detection kit (Enzo), mitoSOX Red mitochondrial superoxide indicator (Molecular probes) and ATP determination kit (Molecular Probes), MitoTEMPO (Sigma), TREM-1 agonist antibody (R&D).

### Murine Models of Renal IR

All animal experiments were approved by the Institutional Animal Care and Use committee of the University of Amsterdam and were in compliance with the ARRIVE guidelines (NC3Rs). Eight-week-old male C57Bl/6J (B6J) mice were purchased by Charles River. TREM1/3 double KO mice were generated, as previously described (18), backcrossed to a C57Bl6 background and bred in the animal facility of the Academic Medical Center in Amsterdam, The Netherlands. Age- and sex-matched mice were used. Animals were kept under standard environmental conditions (temperature, humidity, ventilation, light/dark cycle), housed in specific pathogen-free conditions (SPF) with *ad libitum* access to water and food.

Wild-type (WT) C57BL/6 and TREM-1/3 KO mice (n = 8) were subjected to severe AKI (bilateral renal IR, 25 min clamping time) or mild AKI (unilateral renal IR, 20 min clamping time), as previously described (12). Under 2.5% isoflurane-induced anesthesia, mice underwent clamping of the renal artery for the previously mentioned time periods. Upon completing surgery, muscle and skin layers were closed with surgical sutures (6-0, Tyco). Fifty μg/kg buprenorphine was administered through a subcutaneous injection, for analgesic purposes (Temgesic Shering-Plow). Animals were sacrificed via cardiac exsanguination followed by cervical dislocation, at day 1, 5, and 10 post-surgery. Sham mice were sacrificed at day one and used as control for mild AKI experiment. Contralateral kidneys from mild AKI experiment, were used as controls for PKM2 staining.

For the isolation of primary proximal TECs, we used a well-established protocol from our department (19). Briefly, kidneys were removed and stored on ice in Hank's Balanced Salt Solution (HBSS), supplemented with 50 U penicillin and 50 μg/ml streptomycin. After removal of the renal capsule, tissue was minced with scalpels. A single-cell suspension was obtained by means of collagenase IV digestion. Cells were strained over a 70- and 40-μm filter mesh and cultured in HK-2 medium (see below) under standard culture conditions.

### Cell Culture and Hypoxia/Reoxygenation Experiment

Cells were cultured in HK-2 medium (DMEM/F12 medium, supplemented with 10% FBS, 5 μg/ml insulin and transferrin, 5 ng/ml sodium selenite, 20 ng/ml triiodo-thyrionine, 50 ng/ml hydrocortisone, and 5 ng/ml prostaglandin E1 with l-glutamine and antibiotics). IM-TECs were cultured at 33°C in the presence of 10 ng/ml IFN-γ (ProSpec) and kept at 37°C without IFN-γ for another week before the start of the experiment (20). Primary TECs were isolated and cultured as described above (21).

Hypoxia/reoxygenation experiments were performed as follows. Cells (IM-TECs or primary TECs) were cultured until confluency, incubated in complete medium in the HypOxystation H35 (Don witley scientific) and cultured for 2 days in 1% Oxygen, 5% CO2 at 37°C, in order to mimic the hypoxic condition. Cell viability was assessed with the MTT staining (data not shown). Normoxic cells were kept in culture under standard conditions. After 2 days of hypoxia, both hypoxic and normoxic cells received fresh medium and were cultured for an additional 24 h under standard conditions (re-oxygenation). MitoTEMPO (100μM) or TREM-1 agonist (mTREM-1: 10μg/ml) were added in the culture media during the re-oxygenation time (24 h).

### *In vitro* Assays

All *in vitro* assays were performed in primary TECs from WT and TREM-1/3 KO mice (n = 5–7) and 5–6 replicates were examined per animal.

Cellular oxygen consumption rate (OCR) was determined in primary and immortalized TECs plated on a PureCol coated XF 96 well assay plate, one day prior to the assay. OCR data were determined with the XFe96 Extracellular flux analyzer (seahorse bioscience) using the XF Cell Mito Stress Test which was used according to the provided protocols. Briefly, cells were incubated in assay medium (DMEM phenol red, 25 mM D-glucose, 1 mM Na pyruvate, 2 mM glutamine, adjusted to pH 7.4) at 37°C in a non-CO2 incubator for 1 h prior to metabolic measurement. During the metabolic assay, oxygen consumption rates were measured over time at basal level and after the consequential addition of oligomycin (1.5 μM), FCCP (1 μM), and rotenone (1.25 μM) together with antimycin A (2.5 μM). The indicated compound concentrations are final concentration in assay medium. OXPHOS parameter calculations are according to the Mito stress test provided by Agilent. The following parameters were assessed as described: basal respiration (difference between basal OCR and OCR after addition of oligomycin A+rotenone), ATP-linked respiration (difference in OCR before and after oligomycin addition), maximal respiration (OCR after FCCP minus OCR after oligomycin A+rotenone), spare respiratory capacity (difference between maximal and basal OCR), non-mitochondrial respiration (OCR rate after last injection of oligomycin A+rotenone), and H+ (proton) leak (difference in OCR after oligomycin addition and non-mitochondrial respiration). After the run, supernatant was carefully aspirated and crystal violet cell staining was performed to normalize the values based on cell input. Cells were stained with 0.4% crystal violet in methanol, washed with deionized water, and eluted with methanol. Absorbance of crystal violet was measured at 570 nm (19).

For ATP cell measurements, primary TECs were subjected to hypoxia/reoxygenation. ATP levels were detected with a kit, according to the manufacturer's protocol and adjusted for cell input by total protein concentration, as determined by BCA.

To examine the effect of hypoxia/reoxygenation on mitochondrial superoxide production, primary TECs were labeled with 5 μM MitoSOX Red in HBSS, supplemented with calcium and magnesium (Gibco), for 10 min in the dark at 37°C. Relative fluorescence was measured by FACS.

For the measurement of mitochondrial membrane polarity, primary TECs were plated in black 96-well Clear plates (Greiner) and subjected to hypoxia/re-oxygenation experiments as described above. Mitochondrial polarity was determined using the MITO-ID Membrane potential cytotoxicity kit (Enzo Life Sciences), which uses a cationic dual-emission dye. The dye emits green fluorescence at ~530 nm when present in the cytosol as a monomer; once the dye enters polarized mitochondria, it forms aggregates which shift the emitted light from 530 to 570 nm. Dual fluorescence was measured using the CLARIOstar plate reader. Data are expressed as the ratio between the red fluorescence (mitochondria) (514 nm Ex/590 nm Em) and green fluorescence (cytoplasm) (514 nm Ex/529 nm Em), as previously described (22).

### Flow Cytometry Analysis (FACS)

To determine TREM-1 expression on proximal TECs, cells were detached by treatment with trypsin, washed and suspended in staining buffer (PBS without Ca2+ Mg2+, 0.5% BSA, 2 mM EDTA, 0.025% NaN3). Trypsin digestion did not affect TREM-1 surface expression, as observed in blood-derived granulocytes (data not shown). Anti-CD16/32 (clone 24G2) was added for 10 min to prevent non-specific, Fc-mediated, binding. Cells were then incubated with biotinylated-anti-TREM-1 Ab (gift from Colonna lab), followed by staining with streptavidin-APC. Propidium Iodide was added prior to acquisition for dead/live staining. Samples were analyzed using the FACSCanto II flow cytometer (BD Biosciences) and data were analyzed using FlowJo 7.6 software (TreeStar).

### SA-β-Galactosidase

Microscopy: frozen kidney tissue or cells cultured on coverslips were used for microscopic analysis. Fixation and staining was performed with the SA-β-Gal staining kit, according to the manufacture's protocol. Senescent cells were identified as blue-stained cells by standard light microscopy. Quantification of positive staining was performed with Image Pro Premier (vs. 9.3; Media Cybernatics, Rockville, MD, USA). We used the smart segmentation approach to segment the immunopositive area, which is expressed as a percentage of the total tissue surface (area). The percentage of the immunopositive area was calculated in images of 10 non-overlapping HPFs, per slide (*n* = 5).

Activity: SA-β-Gal activity was measured by means of a flow cytometric assay, using the fluorogenic substrate C_12_FDG (Invitrogen), as previously described (23). Briefly, cells were previously incubated with Fixable Viability Stain 780 in order to exclude dead cells, followed by incubation with Bafilomycin at 37°C for 1 h. β-gal substrate was incubated for another hour at 37°C. Cells were washed with PBS and acquired with a FACS Canto II (BD).

### Determination of Intracellular Metabolite Abundance by LC-MS (Metabolomics)

Primary TECs isolated from WT and TREM1/3 KO mice (n = 6/5), seeded at equal density and cultured for 72 h, were used for metabolomics experiments, which were performed at the Metabolomics Core Facility of the Academic Medical Center, University of Amsterdam. Procedure is explained below.

Cells were washed with ice-cold PBS and metabolism was quenched by adding 1 mL of ice-cold methanol followed by 1 mL of ice-cold water. Cells were collected by scraping. The samples were incubated in a sonication bath and sonicated for 15 min. The homogenate was transferred to a 2 mL tube and 1 mL of chloroform was added, after which the homogenate was vortexed and centrifuged for 5 min at 14,000 rpm at 4°C. The “polar” top layer was transferred to a new 1.5 mL tube and dried in a vacuum concentrator. Dried samples were dissolved in 100 μL methanol/water (6/4; v/v).

For the analysis, a Thermo Scientific ultra-high pressure liquid chromatography system (Waltman) coupled to Thermo Q Exactive (Plus) Orbitrap mass spectrometer (Waltman) was used. The autosampler was held at 10°C during the runs and 5 μL sample was injected onto the analytical column. The chromatographic separation was established using a SeQuant ZIC-cHILIC column (PEEK 100 x 2.1 mm, 3.0 μm particle size, Merck) and kept at 15°C. The flow rate was 0.250 mL/min. The mobile phase was composed of (A) 9/1 acetonitrile/water with 5 mM ammonium acetate; pH 6.8 and (B) 1/9 acetonitrile/water with 5 mM ammonium acetate; pH 6.8. The LC gradient program was as follows: beginning with 100% (A) hold 0–3 min; ramping 3–24 min to 20% (A); hold from 24 to 27 min at 20% (A); ramping from 27 to 28 min to 100% (A); and re-equilibrate from 28 to 35 min with 100% (A). The MS data were acquired in negative mode at full scan range at 140,000 resolution. Interpretation of the data was performed in the Xcalibur software (Thermo Fisher).

### Wound Healing Assay

Primary TECs from WT and TREM1/3 KO animals (*n* = 5) were isolated, cultured and subjected to *in vitro* IR, as described above. Subsequently, a scratch was made using a p100 pipette tip and cells were monitored for the following 24 h, using a live cell phase-contrast microscope (Leica DMi8). Settings were adjusted with LasX software (Leica). Recovery was assessed by an in-house developed method (CiMicro software), which measures the surface of the wound area (in pixels).

### Stable Gene Silencing by sgRNA

Stable knockout cells were generated by CRISP/Cas9 technology; IM-TECs were transduced with lentiviral particles harboring single guide RNA (sgRNA) for 24 h, in the presence of 8 μg/ml polybrene (Sigma Aldrich). Puromycin (Sigma Aldrich) selection was initiated 48 h after transduction.

Lentiviral particles were produced by transfecting HEK293T cells, using GENIUS DNA Transfection Reagent (Westburg), with the following vectors: pMD2.G/VSVG (Addgene 12259), pPAX2 (Addgene 12260), and pLentiCRISPRv2 (Addgene 52961) at a DNA ratio of 6:15:20 μg DNA ratio, respectively. Single guide RNA (sequence: CTTCCATCCTGTCCGCCTGG) targeting murine TREM-1 was inserted into the lentiviral vector pLentiCRISPRv2, according to the protocol described by Sanjana et al. (24).

### BrDU Incorporation Assay

For Cell-cycle analysis, cells were incubated for 1 h with 20 μM BrdU (Sigma Aldrich) and subsequently fixed in 70% ice-cold ethanol. Cells were processed as described previously in our department (25). Staining with anti-BrdU FITC (clone B44; BD) was performed in buffer, containing 0.05% Tween-20/0.5% BSA in PBS. After washing, cells were stained with 0.1 μM TO-PRO-3-iodide (Invitrogen Life Technologies) in PBS/0.5% BSA, containing 500 μg/mL of RNAse-A (Bioke), for 15 min at 37°C. Cell-cycle distribution was analyzed by flow cytometry.

### Protein Determination by ELISA and Western Blot

The levels of KC, MCP-1, and soluble TREM-1 protein (sTREM-1) were measured by ELISA in kidney homogenates. Plasma levels of sTREM-1 were also measured. Frozen renal tissues were processed in Greenberger lysis buffer (150 mM NaCl, 15 mM Tris, 1 mM MgCl2 pH 7.4, 1 mM CaCl2, 1% Triton x-100), supplemented with 1% protease inhibitor cocktail (Sigma-Aldrich, Zwijndrecht, The Netherlands). Renal TREM-1, KC and MCP-1 levels were adjusted for total protein concentration, as measured by BCA (Thermo Fischer).

For tissue protein isolation, 30 μM—thick, frozen kidney slices were used. Tissue and cells were lysed in RIPA buffer (50 mM Tris pH7.5, 0.15 M NaCl, 2 mM EDTA, 1% deoxycholic acid, 1% NP-40, 4 mM sodium orthovanadate, 10 mM sodium fluoride), supplemented with 1% protease inhibitor cocktail (Sigma-Aldrich). Lysates were loaded onto a 4–12% Nupage gel and blotted onto a PVDF membrane. After blocking aspecific signal, blots were incubated overnight with primary antibodies. Blots were incubated with horseradish-peroxidase-conjugated secondary antibodies and detected with ECL (Pierce).

### Histology, (Immuno)-Histochemistry, and Microscopy

Paraffin-embedded kidney tissue, was fixed in 4% formalin for 24 h before processing. For (immuno) histological examination, 4 μM-thick paraffin sections were used. The tubular injury score was determined in PAS-D-stained sections, by a pathologist, in a blinded manner, using a 5-point scale that is based on the presence of necrosis, as previously described (26). Immunohistochemical staining was performed for the detection of macrophages, myofibroblasts, collagen deposits, glycolysis, proliferating, and apoptotic TECs. Sections were processed for antigen retrieval followed by overnight incubation with primary antibodies, at 4°C. The following day, slides were incubated with peroxidase-conjugated secondary antibodies for 30 min and stained with 3,3-diaminobenzidine (DAB). The amount of positive staining for F4/80, αSMA, collagen I and PKM2 was quantified with ImageJ software, Fiji (NIH), a computer-based imaging analysis for reliable biomarker quantitation. Proliferating TECs were counted in the cortico-medullary region in 10 randomly chosen non-overlapping HPFs (magnification 20X). For fluorescent microscopy, cells cultured on coverslips were used. Primary and secondary antibody labeling was performed in 0.5% BSA and 0.1% Triton X-100. Secondary antibodies were detected with either Texas Red or Alexa fluor-labeled antibodies. Cells were counterstained with Hoechst 33342 for nuclear staining (Sigma). Fluorescence microscopy and image acquisition was performed on a Leica DM5000B using the LAS acquisition software (Leica). Percentage of pH3 nuclei was determined by counting total and pH3 positive nuclei in a blinded fashion.

### RT-PCR

Total RNA was extracted from frozen kidneys using Trizol-reagent (Sigma-Aldrich). cDNA was synthesized using M-MLV reverse transcriptase and oligo-dT primers. Transcript analysis was performed by real-time quantitative PCR on the Roche Light Cycler 480 using SYBR green master mix (Bioline). Relative expression was analyzed using LinRegPCR (developed by Hearth failure research center, University of Amsterdam, the Netherlands). Gene expression was normalized to murine Peptidylprolyl Isomerase A (*Ppia*) and Tata Box binding Protein (*Tbp*) housekeeping genes. Murine primer sequences are listed in Supplementary Table 1.

### Statistics

For *in vivo* experiments, comparisons between 2 groups were analyzed using Mann-Whitney U-test. Comparisons between more than 2 groups were analyzed with the Kruskal-Wallis test followed by Dunn's multiple comparisons test. Survival curves were analyzed by the Log-rank (Mantel-Cox) test. For *in vitro* experiments, comparisons between two groups were analyzed using the two-tailed student's *t*-test. Values are expressed as mean ± standard error of the mean (S.E.M). *P* < 0.05 were considered statistically significant.

## Results

### TREM1/3 Limits Maladaptive Renal Repair

**Figure 1:**
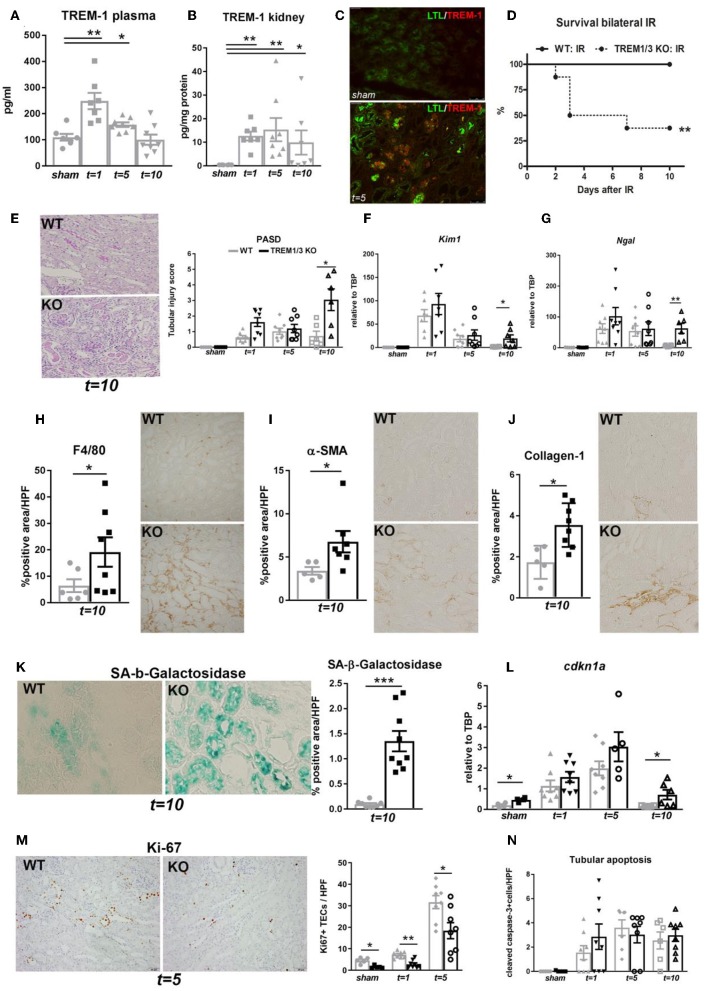
TREM-1 limits the maladaptive repair following renal IR injury. **(A)** Plasma TREM-1 concentration and **(B)** TREM-1 protein levels in kidney lysates from sham-operated mice and WT mice subjected to bilateral IR (25 min clamping) and sacrificed at day 1, 5, and 10 post-surgery. **(C)** Representative pictures of immunofluorescence staining on kidney tissue from WT mice in sham-operated conditions and 5 days post-IR, with TREM-1 (red) and *lotus tetragonolobus lectin* (LTL: proximal tubule marker) in green. **(D)** Survival graph of WT and TREM1/3 KO mice subjected to bilateral IR for 25 min (= severe AKI). **(E)** Quantification of tubular injury obtained by histopathological scoring of PAS-D-stained, paraffin-embedded, renal sections from WT and TREM1/3 KO animals sacrificed at day 1, 5, and 10 post-unilateral IR (= mild AKI). Representative pictures of WT and TREM1/3 KO animals at day 10 post-IR. **(F,G)** Renal expression of tubular injury markers *Kim1* and *Ngal* measured by RT-PCR in tissue isolated from sham WT, WT and TREM1/3 KO mice sacrificed 1, 5, and 10 days post-IR. **(H)** Quantification of immunohistochemical staining and representative pictures for macrophages (F4/80), **(I)** myofibroblasts (α-SMA) and **(J)** collagen type-1 deposits in WT and TREM1/3 KO mice 10 days post-surgery (magnification 40X). **(K)** Quantification and representative pictures of SA-β-Gal staining in WT and TREM1/3 KO mice 10 days post-surgery (magnification 40X). **(L)** Transcript expression of renal *cdkn1a* (p21) in WT and KO mice in sham conditions and post-IR. **(M)** Quantification of TECs proliferation detected by Ki67 immunohistochemical staining in WT and TREM1/3 KO mice in sham conditions and 1 and 5 days post-surgery. Representative pictures of WT and TREM1/3 KO kidneys at day 5 (magnification 20X). **(N)** Quantification of tubular apoptosis detected by cleaved-caspase-3 immunohistochemical staining in WT and TREM1/3 KO mice in sham conditions and post-IR. Data are expressed as mean ± SEM. **(A,B)** Kruskal–Wallis and Dunn's multiple comparison test, **(D)** Log-rank (Mantel-Cox) test. **(E–N)** One way Anova followed by Mann–Whitney test were used to determine statistical differences. ^*^P < 0.05, ^**^P < 0.01, ^***^P < 0.001.

We have previously described TREM-1 increase in plasma and kidney 1 day post-bilateral clamping of renal artery, which induces a severe AKI response (12). Here, we show that TREM-1 levels in plasma increase as well 5 days post-bilateral IR (Figure 1A). Additionally, renal TREM-1 expression increased during the recovery (*t* = *5*) and resolutive phases (*t* = *10*) as compared to sham kidneys (Figure 1B). Since TREM-1 protein remains highly expressed during active repair (*t* = *5*) and that TREM-1+ cells co-localize with *Lotus tetragonolobus* lectin (LTL), a proximal tubular marker (Figure 1C), we further investigated the role of TREM-1 in tissue repair. We adopted a murine model of genetic deletion which simulate the human TREM-1 system regulation, where *TREM3* appears to be a pseudogene: the TREM1/3 double KO mice, which underwent bilateral IR together with WT animals.

TREM1/3 KO animals displayed increased mortality during the recovery phase compared to WT mice (Figure 1D), although renal function and inflammatory parameters, such as keratinocyte chemoattractant (KC) and monocyte chemoattractant protein-1 (MCP-1) (Table 1) were unchanged in both animal strains in sham or day 1-5 post-IR. Infiltration of inflammatory cells such as granulocytes and macrophage was also similar between animal strains (data not shown).

**Table 1:** Renal Inflammation in WT and TREM1/3 KO mice upon severe AKI (*N* = 8 mice per group).

	**KC (pg/mg protein)**		**MCP1 (pg/mg protein)**	
	**WT**	**TREM1/3 KO**	**WT**	**TREM1/3 KO**
Sham	756 ± 104	659 ± 89	22 ± 3	18 ± 2
*t = 1*	1318 ± 153	1635 ± 90	27 ± 2	34 ± 4
*t = 5*	831 ± 174	1432 ± 379	54 ± 12	71 ± 21^&^

*KC and MCP-1 levels were measured by ELISA. Values are expressed as mean ± SEM.^&^N = 5 mice/group. The t = 10 time point was not included in this table as the number of surviving KO animals was significantly decreased compared to that of WT mice (WT: n = 8 vs. TREM1/3 KO: n = 3)*.

To avoid mortality, we switched to a milder model of AKI (unilateral clamping of renal artery for 20 min). We observed that, compared to WT animals, mice lacking TREM1/3 displayed increased tubular damage 10 days post-IR, through histopathological assessment of PAS-D-stained sections (Figure 1E and Supplementary Figure 1A) and evaluation of tubular injury markers, such as *Kim-1* and *Ngal* (Figures 1F,G). As this is indicative of impaired repair that could eventually lead to renal fibrosis (27), we looked at renal macrophage infiltration, well-known to facilitate tubular repair but, if persistent, may further boost inflammation and drive fibrosis (28). Renal tissue from TREM1/3 KO mice showed increased infiltration of F4/80+ macrophages, compared to WT animals at *t* = *10* (Figure 1H). The excessive macrophage infiltrate was also associated with the development of fibrosis, as reflected by an increase in α-SMA+ myofibroblasts and collagen type-1 deposits, detected also by picro-sirius red (PSR) (Figures 1I,J and Supplementary Figure 1B). Besides these detrimental effects, absence of TREM1/3 also resulted in impaired tubular regeneration due to the accumulation of senescent cells, as shown by SA-β-Galactosidase+ TECs, a common marker for senescence (29) (Figure 1K). In line, transcript levels of cyclin-dependent kinase inhibitor 1A (*Cdkn1a*/p21), which is upregulated in cell-cycle arrest and senescence (6), appear to be increased in KO animals at *t* = *10* as well as in sham-operated mice, when compared to WT animals (Figure 1L). This is likely to be linked to the decreased TECs proliferation observed in KO animals, detected by Ki67 staining, already in sham conditions but also after IR (Figure 1M). Tubular apoptosis is also associated with fibrosis in the kidney, but cleaved-caspase-3+ TECs revealed no differences between WT and KO animals post-IR (Figure 1N), suggesting that apoptosis is not involved in this maladaptive process. Taken together, these *in vivo* evidences suggest that the TREM1/3 deficiency results in a maladaptive repair after AKI.

### TREM1/3-Deficient TECs Display G2/M Arrest and Impaired Energy Metabolism

**Figure 2:**
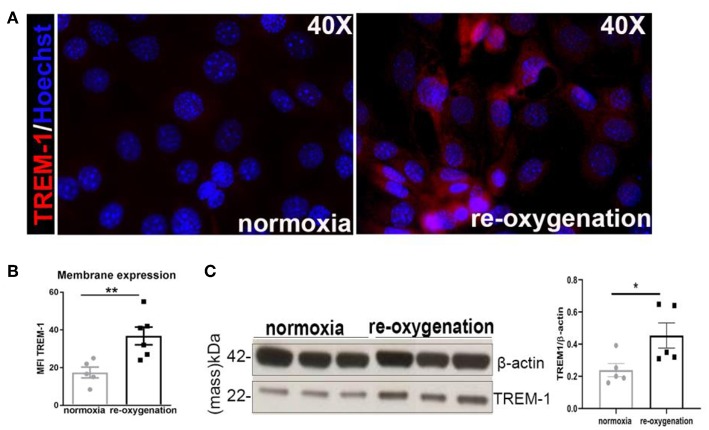
TREM-1 is expressed in TECs upon hypoxia-reoxygenation **(A)**. Representative immunofluorescent images of IM-TECs cultured on coverslips in normoxia conditions and after hypoxia-reoxygenation. Cells were stained with TREM-1 (Alexa 594, red) and Hoechst (nuclei). **(B)** Mean Fluorescence intensity (MFI) of TREM-1 membrane expression measured by flow cytometry. **(C)** Protein lysates of the same experiment blotted for anti-TREM-1 Ab and β-actin. In **(B,C)** data are expressed as mean ± SEM and the unpaired *t*-test was used to determine statistical differences. *N* = 5–6 animals/group. ^*^P < 0.05, ^**^P < 0.01.

Since we observed an overall decrease in TECs proliferation in KO animals and given that tubular proliferation is essential for regeneration post-IR (3, 4), we aimed to investigate TREM1/3's role in tubular response after IR. Therefore, we evaluated TREM-1 expression in TECs following the *in vitro* hypoxia/reoxygenation injury, to model the hypoxic damage experienced by the TECs during IR. TREM-1 increases in TECs during re-oxygenation compared to normoxic cells (Figures 2A–C), suggesting a role for TREM-1 in TEC's response to hypoxic damage.

**Figure 3:**
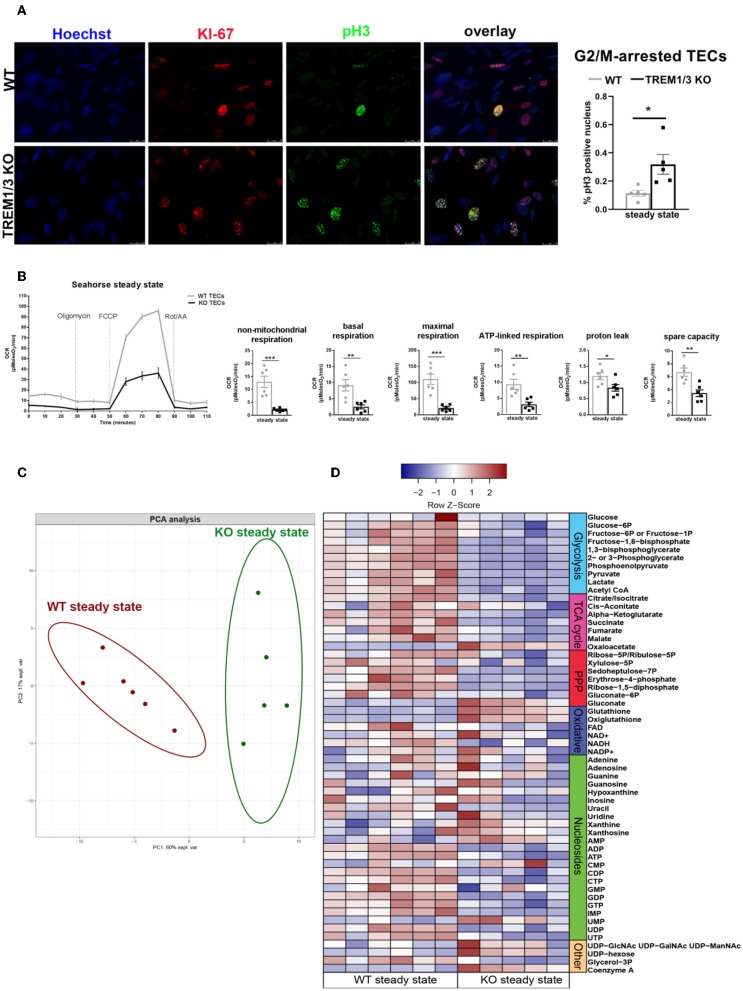
TREM1/3 KO primary TECs display arrest in G2/M and reduced mitochondrial metabolism at steady state. **(A)** Representative immunofluorescent images of primary TECs at steady state isolated from WT and TREM1/3 KO animals (*n* = 5) (magnification 40X). Cells were stained with Hoechst (nuclei), anti-Ki67 (proliferating cells), Alexa 594 and anti-phosphorylated histone H3(ser10)- Alexa 488-conjugated (to identify cells in G2/M). Quantification of percentage of phosphorylated histone H3 positive nuclei per HPF. **(B)** Seahorse Bioanalyzer OCR readout of a typical mitochondrial function assay using WT and TREM1/3 KO primary TECs without adjustment for cell number. AA, antimycin A; rot, rotenone. Conditions were measured in triplicate. The bar graphs represent data adjusted for cell input for WT and TREM1/3 KO primary TECs. **(C)** Principal component analysis (PCA) of intracellular metabolite levels from WT and TREM-1/3 KO primary TECs at steady state (CTR) as determined by LC-MS (metabolomics) (*n* = 5/6 mice per group). **(D)** Differences in metabolic pathways are displayed by the heatmap. **(A,B)** All data are expressed as mean ± SEM and the unpaired *t*-test was used to determine statistical differences. ^*^P > 0.05, ^**^P < 0.01, ^***^P < 0.001.

The *in vivo* study also revealed a decreased proliferation of TECs in KO animals already in sham conditions. Consistent with this observation, an increased percentage of phosphorylated Ser-10 of histone H3 (pH3) nuclei was detected in TREM1/3 KO primary TECs at steady state, indicative of G2/M-arrest (Figures 3A, 6). Metabolically, cells rely more on oxidative phosphorylation (OXPHOS) than glycolysis to enter into mitosis (30). We, therefore, examined the oxygen consumption rate (OCR) using the Seahorse respirometer. TECs lacking TREM1/3 showed a significant down-regulation in oxidative metabolism, as both basal and non-mitochondrial respiration levels were decreased. Accordingly, the lack of TREM1/3 impaired the ability to maximize OXPHOS usage and cope with sudden high-energy demand given by the mitochondrial respiration uncoupler FCCP and by the lower spare capacity. In addition, the OCR rate associated with ATP production, calculated after administration of the ATP synthase inhibitor oligomycin, was also reduced in TREM1/3 KO TECs (Figure 3B). Similar results were obtained using an immortalized TEC cell line where only TREM-1 expression was silenced by CRISPR/Cas9 technology. Absence of TREM-1 solely resulted in delayed G2/M progression and reduced mitochondrial respiration (Supplementary Figures 2A,B). These results suggest that even if TREM-3 may play a similar role in tubular proliferation and oxidative metabolism, this might not be so relevant. To directly demonstrate the impact of TREM1/3 deficiency on TEC metabolism, we performed mass spectrometry analysis of cellular metabolites. TREM1/3 absence has an important effect on tubular energy metabolism in general, as shown by the dissimilarities in metabolite content distribution in the PCA 2D score plot (Figure 3C). As displayed in the heatmap (Figure 3D), lack of TREM1/3 also results in an overall reduction in key intermediates of the glycolytic, tricarboxylic acid (TCA) and pentose phosphate pathway (PPP). Thus, the effect of TREM1/3 absence, compared to WT cells, may not be restricted to OXPHOS. Interestingly, the levels of the antioxidants glutathione (GSH) and oxiglutathione (GSSG) were increased in KO TECs suggesting an enhanced activity of mitochondrial defense systems against reactive oxygen species (ROS) accumulation (Figure 3D).

Hence, these data suggest that TREM-1 may be an immune-sensor that controls energy metabolism and promotes TEC proliferation.

### TREM1/3 Promotes Tubular Homeostasis After Hypoxia, by Preserving Mitochondrial Integrity

**Figure 4:**
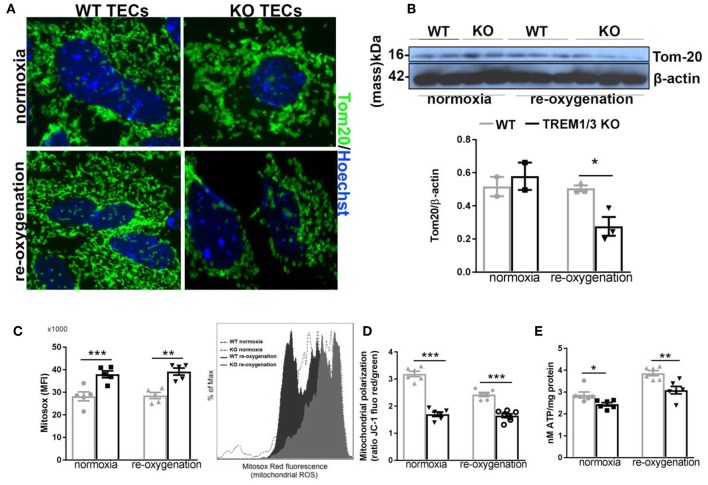
TREM1/3 deficient TECs display an altered mitochondrial homeostasis. **(A)** Representative immunofluorescent images of primary TECs stained for mitochondrial protein TOM20 (Alexa488) and Hoechst (nuclear staining), under normoxic conditions and 24 h after re-oxygenation (magnification 100X) (*N* = 3 animals per conditions). **(B)** Western blot of WT and TREM1/3 KO primary TECs blotted for TOM20 and β-actin. **(C)** Mitochondrial ROS production measured by FACS analysis with MitoSox probe. Histogram representation of Mitosox events expressed as % of max. (*N* = 5 animals per group). **(D)** Mitochondrial polarization measured by cationic dual-emission fluorescent probe, expressed as a ratio between red (mitochondria, J-aggregates) and green (cytoplasm, dye monomers) fluorescence intensity. Results are expressed as a ratio of red and green fluorescence emission. (*N* = 5 animals per group). **(E)** ATP production in protein lysates of WT and TREM1/3 KO primary TECs, corrected for the total protein concentration measured by BCA (*N* = 5/6 animals per group). All data are expressed as mean ± SEM and the unpaired *t*-test was used to determine statistical differences. ^*^P < 0.05, ^**^P < 0.01, ^***^P < 0.001.

From previous evidences obtained in bone marrow-derived macrophages, we know that TREM-1 plays a role in mitochondrial integrity (17). Additionally, the increased antioxidant levels detected in TREM1/3 KO TECs suggest an impaired control of redox balance, which could lead to higher ROS levels and impact mitochondrial energy metabolism (31). An in-depth analysis of mitochondria following the hypoxia/re-oxygenation model we adopted *in vitro*, revealed an aberrant mitochondrial homeostasis in TREM1/3 KO TECs. To gain more insight into the morphology, we looked at the expression of the translocase of outer membrane 20 (TOM20), a mitochondrial marker, which clearly showed rounded and fragmented mitochondria in TREM1/3 KO TECs, as compared to the typical filamentous network of mitochondria observed in WT cells, already in normoxic conditions. This altered morphology worsened during re-oxygenation conditions (Figure 4A) and was also displayed by WB analysis, where a decrease in protein content was evident in KO primary TECs (Figure 4B).

TREM1/3 deficiency was also associated with increased mitochondrial superoxide production, detected by the Mitosox probe, both in normoxic and hypoxia/re-oxygenation conditions (Figure 4C). Further analysis of mitochondrial function revealed an impressive mitochondrial depolarization, detected with a dual-fluorescence emission mitochondrial membrane potential-sensor (Figure 4D). Consequently, the mitochondrial dysfunction was associated with a decrease in ATP production in TREM1/3 KO TECs, compared to WT cells, under both experimental conditions (Figure 4E). *Pgc1a, Tfam*, phospho-AMPK and Mitofusin-2 expression was found to be similar in WT and TREM1/3 KO TECs, indicating that the impaired mitochondrial homeostasis was not associated with any differences in mitochondrial biogenesis or dynamics (Supplementary Figures 3A–C).

Altogether, these data suggest that the innate immune receptor TREM1/3 preserves mitochondrial homeostasis in TECs, possibly by limiting ROS production.

### Hypoxia-Induced Oxidative Stress Induces a Senescent Phenotype in TREM1/3 KO Primary TECs

**Figure 5:**
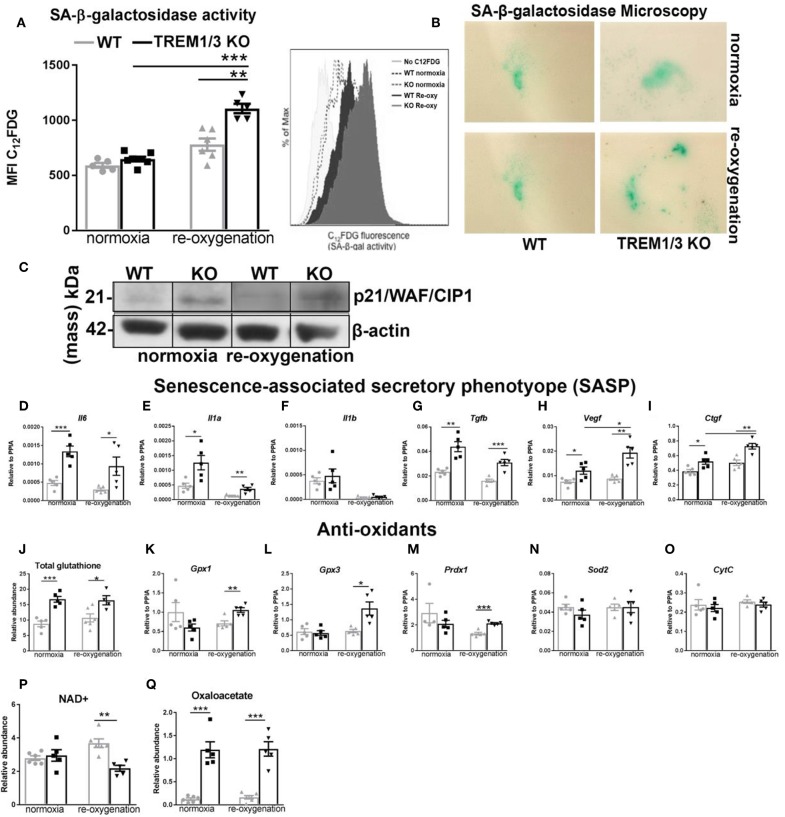
TREM1/3 KO TECs display senescence upon hypoxia-derived oxidative stress. **(A)** FACS analysis of senescence- associated β-galactosidase (SA-βgal) assay by C_12_FDG incorporation in WT and TREM1/3 KO primary TECs in normoxia conditions and upon hypoxia/re-oxygenation (*N* = 5/6). **(B)** (SA-βgal) staining obtained in primary WT and TREM1/3 KO TECs in normoxia and hypoxia/re-oxygenation, cultured on coverslips. Blue/green cells represent senescent cells. **(C)** p21 protein expression shown by western blot in primary TEC lysates isolated from WT and TREM1/3 KO animals during normoxia and hypoxia-reoxygenation. **(D–F)** Transcript expression of SASP-components such as pro-inflammatory (*Il6, Il1a, Il1b*) and **(G–I)** profibrotic genes (*Tgfb, Vegf*, and *Ctgf*) in WT and TREM1/3 KO primary TECs under normoxia/re-oxygenation conditions (*N* = 5). **(J–O)** Anti-oxidant expression, such as total glutathione, measured by metabolomics, and transcript expression of glutathione peroxidase 1-2 (*gpx1* and *gpx3*), peroxiredoxin 1 (*prdx1)*, and cytochrome C *(CytC)*, measured by RT-PCR in WT and KO TECs in normoxia/re-oxygenation conditions (*N* = 5). **(P,Q)** Total NAD+ and oxaloacetate levels measured by metabolomics. All data are expressed as mean ± SEM and the unpaired *t*-test was used to determine statistical differences. ^*^P < 0.05, ^**^P < 0.01, ^***^P < 0.001.

ROS-induced mitochondrial dysfunction can contribute to the development of senescence. With this in mind, we observed tubular senescence *in vivo* after IR and checked whether TREM1/3 KO TECs subjected to hypoxia-reoxygenation could become senescent. By FACS analysis, we looked at C_12_FDG-positive cells (a fluorescent substrate for β-galactosidase, only retained in senescent cells). The oxidative stress induced by the hypoxic conditions, drove only TREM1/3 KO TECs into a senescent state, as shown by FACS and microscopic analysis (Figures 5A,B). Additionally, TREM1/3 KO TECs displayed increased levels of p21^WAF1/CIP1^ protein, under both normoxia and re-oxygenation (Figure 5C), suggesting that the effects of mitochondrial dysfunction and ROS production in KO cells may be mediated through p21. G2/M-arrested and senescent TECs release inflammatory and pro-fibrotic mediators, components of the senescence-associated secretory phenotype (SASP), to further exacerbate renal inflammation and fibrosis (6, 32). Accordingly, already under normoxic conditions, KO TECs displayed an increased secretory phenotype, characterized by higher transcription of pro-inflammatory and pro-fibrotic genes, including *Il6* and *Il1a, Tgfb, Vegf* , and *Ctgf* compared to WT TECs (Figures 5D–I). To investigate whether increased ROS production was the root of senescence, we measured several antioxidants. The relative abundance of total glutathione was actually increased in KO TECs in both experimental conditions, whereas the gene expression of glutathione peroxidase 1-3 (*Gpx1-3*) and peroxiredoxin-1 (*Prdx1*) only increased in KO TECs during re-oxygenation. Superoxide dismutase 2 (*Sod2*) mRNA levels, instead, were unchanged, suggesting that detoxification of hydrogen peroxide, but not ${\text{O}}_{2}^{-}$, is affected in KO TECs (Figures 5J–N). Additionally, no change in *Cytc* were measured between WT and KO cells (Figure 5O). This, together with the absence of differences in caspase-3+TECs *in vivo*, may exclude a role for TREM1/3 in ischemic/oxidative stress-induced apoptosis.

It is likely that the mitochondrial dysfunction in KO TECs have impacted the response to IR in the *in vivo* study. Indeed we found that kidneys from KO animals show a metabolic reprogramming toward glycolysis, assessed by the increased expression of the glycolytic pyruvate kinase isozymes M2 (PKM2) protein, both in contralateral and ischemic kidney that failed to regenerate (Supplementary Figure 4A). To further support TREM-1's protective role against senescence, ROS generation and mitochondrial damage, we used another *in vitro* model of oxidative stress, where we exposed primary WT and KO TECs to the antineoplastic agent Doxorubicin (DOX), which was previously reported to induce both senescence and mitochondrial damage with increased ROS production (33). To avoid cell death (data not shown), we treated TECs with a low concentration of DOX (0.1 μM). Similar to the result obtained with the hypoxic damage, lack of TREM1/3 disrupted mitochondrial morphology (TOM20) (Supplementary Figure 5A), increased intracellular ROS (Supplementary Figure 5B) and induced senescence markers (β-gal, p21, SASP) (Supplementary Figures 5C–E), with no effect on mitofusin-2 expression nor AMPK activation (Supplementary Figures 5F,G). This data suggest a general role for TREM1/3 in preventing cellular senescence and excessive ROS accumulation.

Mitochondria metabolites are also impaired during senescence. Previous reports have linked decreased NAD^+^ levels with senescence and mitochondrial dysfunction (34). Thus, we assessed total NAD^+^ content in TECs and found significantly decreased NAD^+^ levels in KO TECs following hypoxia/re-oxygenation (Figure 5P). This nicely correlates with the increased β-galactosidase activity associated with senescence in KO cells (Figure 5A). Besides NAD+, other metabolites of the TCA cycle can also influence cellular function. Interestingly, we found that TREM1/3 KO TECs display an aberrant accumulation of oxaloacetate (OA), a pivotal regulator of the metabolic flux through the TCA cycle, whose concentration should be tightly controlled within the mitochondrial matrix to avoid growth arrest (35) (Figure 5Q).

### TREM-1 Modulates “Stress-Induced Senescence” in TECs

**Figure 6:**
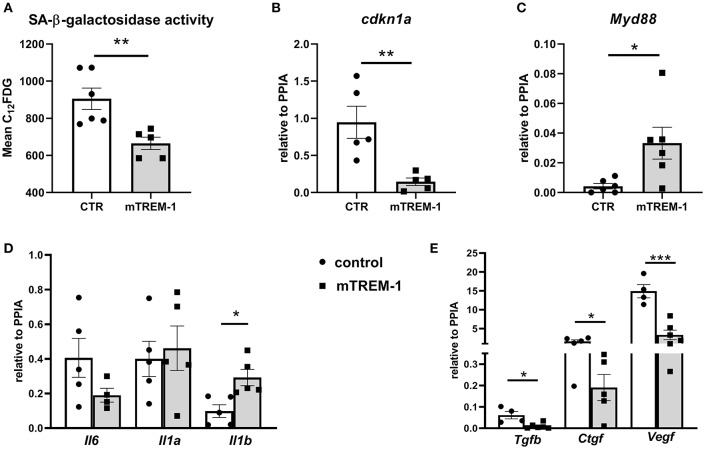
TREM-1 signaling modulates senescence in TECs. **(A)** FACS analysis of senescence- associated β-galactosidase (SA-βgal) assay by C_12_FDG incorporation in WT primary TECs stimulated with isotype control (CTR) and 10 μg/ml TREM-1 agonistic antibody (mTREM-1), during re-oxygenation conditions (24 h) (*N* = 5/6 animals per group). **(B-C)** Relative expression of p21 (*cdkn1a*) and *Myd88* genes in WT TECs in CTR and mTREM-1 conditions, during reoxygenation. **(D)** Transcript expression of SASP-components such as pro-inflammatory (*Il6, Il1a, Il1b*) and **(E)** profibrotic genes (*Tgfb, Ctgf, and Vegf*) in WT TECs under re-oxygenation conditions stimulated with isotype control and mTREM-1 (*N* = 5–6 animals/group). All data are expressed as mean ± SEM and the unpaired t-test was used to determine statistical differences. ^*^P < 0.05, ^**^P < 0.01, ^***^P < 0.001.

Next, we investigated whether TREM-1 signaling can modulate the senescence phenotype after hypoxia/re-oxygenation. TREM-1 ligation by agonistic antibody during re-oxygenation conditions (24 h) ameliorates the SA-β-galactosidase activity (Figure 6A) as well as p21 gene expression (*cdkn1a*) (Figure 6B) and increased *Myd88* gene expression (Figure 6C). As another feature of senescence is the SASP, we looked at the pro-inflammatory and pro-fibrotic gene expression. TREM-1 ligation did not have any effect on pro-inflammatory SASP (Figure 6D) but was able to ameliorate pro-fibrotic gene expression (Figure 6E).

**Figure 7:**
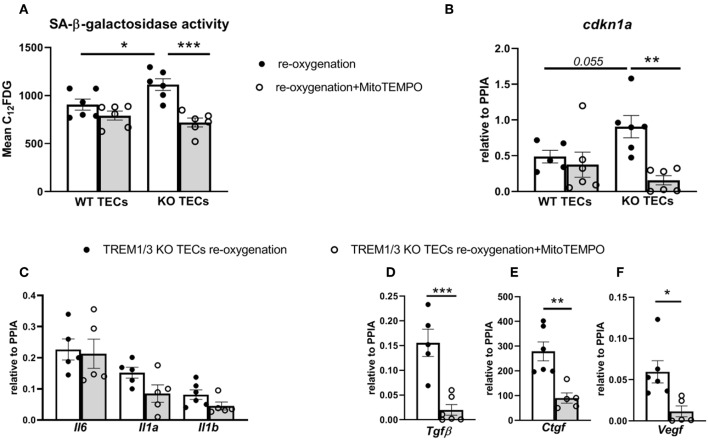
MitoTEMPO partly prevented stress-induced senescence in TREM1/3 KO TECs. **(A)** FACS analysis of senescence- associated β-galactosidase (SA-βgal) assay by C_12_FDG incorporation in WT and TREM1/3 KO primary TECs stimulated MitoTEMPO (100μM) during re-oxygenation conditions (24 h) (*N* = 5/6 animals per group). **(B)** p21 (*cdkn1a*) relative expression after stimulation with MitoTEMPO during reoxygenation in WT and TREM1/3 KO TECs. **(C)** Transcript expression of SASP-components such as pro-inflammatory (*Il6, Il1a, Il1b*) and **(D–F)** profibrotic genes (*Tgfb, Ctgf*, and *Vegf*) in TREM1/3 KO primary TECs under re-oxygenation conditions stimulated with MitoTEMPO (*N* = 5–6 animals/group). All data are expressed as mean ± SEM and the unpaired t-test was used to determine statistical differences. ^*^P < 0.05, ^**^P < 0.01, ^***^P < 0.001.

We reasoned that TREM1/3 KO TECs manifested the so-called “stress-induced” senescence due to an aberrant mitochondrial function, accumulation of ROS and p21 activation (36). To prove this, we stimulated cells during re-oxygenation conditions using a mitochondrially-targeted oxygen radical scavenger, mitoTEMPO. Incubation of TREM1/3 KO TECs in presence of MitoTEMPO completely prevented the SA-β-galactosidase activity and *cdkn1a* gene expression (Figures 7A,B). Additionally, the pro-fibrotic SASP but not the pro-inflammatory SASP were also ameliorated in presence of MitoTEMPO (Figures 7D–F). These data strongly implicate the mitochondrial dysfunction as cause of ‘stress-induced senescence' in absence of TREM-1.

**Figure 8:**
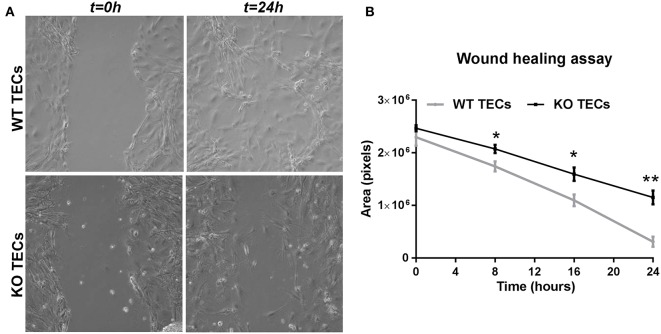
TREM1/3 KO TECs display impaired wound healing capacity after hypoxia-reoxygenation. 24 h after re-oxygenation, the epithelial monolayer of WT and TREM1/3 KO TECs was damaged by a scratch with a p100 pipette tip. **(A)** The damaged area is shown in the pictures taken with a contrast-phase microscope at *t* = 0 and 24 h later. Every 8 h, for up to 24 h, **(B)** the damaged area (shown in pixels) was measured with an in-house software (ci-Micro), developed by the department of cell biology and histology at our institution. All data are expressed as mean ± SEM (*n* = 5) and the unpaired *t*-test was used to determine statistical differences. ^*^P < 0.05, ^**^P < 0.01.

In order to translate these findings into the functional settings of tissue repair, we subjected WT and KO TECs to hypoxia/reoxygenation and then performed a wound healing assay. We measured the wound area (in pixels) at 8, 16, and 24 h after having scratched the monolayer with a pipette tip. In WT TECs the gap was completely closed after 24 h, whereas TREM1/3 KO TECs showed a clear delay in wound healing (Figure 8). This suggests that senescence resulted in impaired wound healing.

Taken together, these data suggest that TREM1/3 deficiency impairs the mitochondrial metabolic flexibility in TECs which resulted in tubular senescence and impacted renal regeneration.

Supplementary Figure 6 shows a schematic representation of our proposed model of TREM1/3-mediated renal repair.

## Discussion

Acute kidney injury (AKI) is a major clinical concern that is considered to be reversible, when not lethal in the acute phase. More recently, however, even mild episodes of AKI have been linked to subsequent development of CKD. The maladaptive repair mechanisms of TECs can be the principal mediator of this chronic progression. Maladaptive tubular cells present senescence, manifested as G2/M arrest and increased SASP. Although innate immune receptors can control cell fate and behavior through regulation of metabolism, a study demonstrating the role of innate immune receptors in the epithelial senescence is still lacking. Herein, we provide novel evidences that the innate immune receptor TREM1/3 mediates senescence through immunometabolism. We extended the knowledge about TREM1/3's function to the control of the mitochondrial metabolic activities and as guardian of the redox balance in tubular cells, which limits senescence. Additionally, this papers highlights a remarkable role for TEC's metabolic flexibility in mediating adaptive renal repair after IR and that might be under the control of innate immune receptors.

TREM-1 does not play a crucial role in the acute phase after AKI (12), whereas in a model of renal fibrosis, might be detrimental due to a direct effect on renal inflammation and M1 macrophages activation (37). However, the role of TREM-1 in renal repair following AKI has not yet been completely unraveled. Herein, we found that renal TREM-1 protein expression is increased 5 days post-IR and that TREM1/3 KO animals subjected to a severe model of AKI show increased mortality during active repair with no differences in inflammatory parameters, such as pro-inflammatory cytokines and granulocytes or macrophages infiltration (data not shown). In a milder model of AKI, TREM1/3 KO animals show no differences in renal damage 24 h post-IR, but do display maladaptive repair with persistent tubular damage, inflammation, fibrosis and tubular senescence. TEC proliferation is crucial for tubular regeneration after AKI, indeed, cell cycle arrest is associated with CKD (38). TREM-1 expression increased in TECs during repair and after hypoxia-reoxygenation injury *in vitro*. Considering that TREM-1 is a hypoxia-inducible gene in DCs and is upregulated in the epithelia of different organs upon inflammation (39–41), altogether these findings suggest a role for TREM-1 in TEC recovery after IR. Nevertheless, we found that TREM1/3-deficiency has profound influence on TECs metabolism and fate already at steady state, resulting in G2/M arrest and decreased energy metabolism. Cells sense and respond to an increase in energy demand for cell cycle progression, but also a metabolic rewiring during hypoxia, is crucial to return to metabolic homeostasis. It appears that TREM1/3 KO TECs due to the metabolic meltdown cannot longer cycle and that the impaired metabolic flexibility leave them unable to cope with the sudden energy deprivation experienced during ischemia. Healthy mitochondria are essential to provide the TECs with an adequate energy supply. TREM1/3-deficient TECs displayed an aberrant mitochondrial homeostasis. The ROS produced by mitochondrial oxidative metabolism are buffered intracellularly by redox cycling systems, such as GSH/GSSG; however, if ROS production exceeds the cell's buffering abilities, oxidative stress and mitochondrial damage can occur (42, 43). It is conceivable that the impaired ability to carry out many metabolic functions observed in the TREM1/3 KO TECs, is due to oxidative damage and that the increased levels of anti-oxidants detected are simply an adaptive response to avoid cell death.

Quiescent TECs rely mostly on oxidative phosphorylation, thus we reasoned that the aberrant mitochondrial phenotype in KO TECs could lead to a metabolic reprogramming. Indeed, in the *in vivo* study we observed that KO animals that presented a maladaptive repair with increased TECs senescence, displayed a metabolic reprogramming toward glycolysis, most likely due to mitochondrial pathology, as previously reported in TECs that failed to regenerate (10).

TREM-1 preserves mitochondrial integrity in bone marrow-derived macrophages, through Mitofusin-2, promoting mitochondrial fusion and inhibiting apoptosis (17). Despite this evidence, no differences in macrophage infiltration were found at basal conditions and early after renal IR, in both severe and mild AKI (data not shown), whereas TECs proliferation was decreased in both severe and mild AKI experiments suggesting that TECs, rather than macrophage infiltration, are most likely responsible for the impaired repair. Additionally, in contrast to previous reports in macrophage, in our model using TECs, mitochondrial dynamics and biogenesis are not affected in TREM1/3 KO TECs (44).

Disturbed mitochondrial homeostasis and excessive ROS production can predispose cells to “stress induces senescence” (42, 43, 45). When TREM1/3 KO TECs were exposed to two different ROS-generating triggers, such as hypoxia/reoxygenation or doxorubucin, this was sufficient to drive the cells into a senescent state. Cell cycle arrest and “stress-induced senescence” involve activation of cyclin-dependent kinases, in order to recognize and repair genetic defects (46). p21 is involved in the G2 checkpoints and its activation is linked to cell cycle arrest and senescence (47). In line with these studies, TREM1/3 KO TECs displayed increased p21 activation in control conditions and after exposure to ROS triggers. Mice deficient for p21 are protected against renal dysfunction and fibrosis after renal ablation (48). Apparently, senescence is important for wound healing but accumulation of senescent cells delays regeneration, just as it happened in our *in vivo* model. Given the fact that a mitochondrial antioxidants (mitoTEMPO) was able to reverse senescence and the pro-fibrotic phenotype in KO TECs, this would further support our hypothesis that TECs in TREM1/3 KO animals display stress-induced senescence which most likely would be the root cause of maladaptive repair and fibrosis. TREM1/3 KO TECs displayed also increased SASP, which could possibly influence the renal microenvironment and create a vicious fibrotic cycle, that hinder renal regeneration and exacerbate fibrosis, as previously described (6). Altogether, these observations indicate that in the absence of TREM1/3, TECs are in G2/M arrest and that IR leads to a permanent growth arrest, manifested as senescence, which results in impaired wound healing.

The profound impact of TREM1/3 absence in TEC's metabolism have been further confirmed by the aberrant accumulation of the mitochondria metabolite oxaloacetate (OA) already at steady state, but also by the NAD+ decline during reoxygenation conditions. The latter might be simply explained by the oxidative stress-induced mitochondrial dysfunction, known to affect this metabolite (34, 49). OA instead, derives from the TCA cycle and participates in the metabolism of energy production and recent research has shown that is affected during cell cycle disturbance. Moreover, OA enhances mitochondrial metabolism by potentiating OXPHOS (50). Thus, accumulation of OA could reflect the decreased flux through the TCA cycle or the attempt of enhancing OXPHOS in TREM1/3 KO TECs.

Despite the fact that we have demonstrated a link between TREM-1 pathway, mitochondrial energy metabolism and senescence, we remain unaware of the exact trigger for TREM-1 activation in this model and how an innate immune receptor present in the plasma membrane can affect mitochondrial homeostasis. Surely, the identification of the TREM-1 ligand in TECs and in the context of IR would help to clarify the mechanism, but this still represents a limitation within TREM-1 research and thus of this study (13).

Finally, this study extends the novel link between innate immunity and tubular metabolism (19), including epithelial senescence. Although it is evident that senescence delays tissue repair and increases the risk of AKI-CKD progression, studies focused on the immunometabolic reprogramming that occurs in tubular senescent cells are at a very early stage. Here, we propose that the innate immune receptor TREM-1, is able to affect mitochondrial function in TECs, which regulates energy metabolism and limits senescence. In this way, TREM-1 might supply TECs with the metabolic flexibility necessary to promote proliferation and regeneration in the tubular epithelium after AKI.

## Data Availability

The datasets generated for this study are available on request to the corresponding author.

## Ethics Statement

All animal experiments were approved by the Institutional Animal Care and Use committee of the University of Amsterdam and were in compliance with the ARRIVE guidelines (NC3Rs).

## Author Contributions

AT, MD, and SF conceived and designed the experiments. AT, AMLS, ER, and CB performed the experiments. NC and LB helped with laboratory techniques. MC provided TREM1/3 KO animals and contributed with background knowledge on TREM-1. AS contributed with knowledge on stress-induced senescence. JL was involved in interpretation of data. AT wrote the manuscript. All authors revised the manuscript and had final approval of the submitted version.

### Conflict of Interest Statement

The authors declare that the research was conducted in the absence of any commercial or financial relationships that could be construed as a potential conflict of interest.
