# Supplementary material for: TREM1/3 Deficiency Impairs Tissue Repair After Acute Kidney Injury and Mitochondrial Metabolic Flexibility in Tubular Epithelial Cells
Source: Front Immunol. 2019 Jul 9;10:1469. doi: 10.3389/fimmu.2019.01469 (PMC6629955; doi:10.3389/fimmu.2019.01469)
Supplement: Supplementary file 1 [file Data_Sheet_1.pdf]

# Supplementary material

# TREM1/3 deficiency impairs tissue repair after acute kidney injury and mitochondrial metabolic flexibility in tubular epithelial cells.

Alessandra Tammaro[1*], Angelique M.L. Scantlebery[1§] , Elena Rampanelli[1§] , Cristiana Borrelli[2,3], Nike Claessen[1], Loes M. Butter[1], Alessandra Soriani[2] , Marco Colonna[4], Jaklien C. Leemans[1], Mark C. Dessing[1&] and Sandrine Florquin[1&].

[1] Amsterdam UMC, Univ(ersity) of Amsterdam, Amsterdam, The Netherlands; [2]Department of Molecular Medicine, Sapienza University of Rome, Laboratory Affiliated with Istituto Pasteur Italia - Fondazione Cenci Bolognetti, Rome, Italy; [3]Center for Life Nano Science, Sapienza, Istituto Italiano di Tecnologia, Rome, Italy. [4]Department of Pathology and Immunology, Washington University School of Medicine, St Louis, Missouri, USA. [§&]Equal contribution

***Corresponding author:**

Alessandra Tammaro, Amsterdam UMC, Univ(ersity) of Amsterdam

Department of Pathology AMC, room L2-112

Meibergdreef 09,1105AZ Amsterdam (the Netherlands)

Telephone number: +31 020 5665715, Email address: a.tammaro@amsterdamumc.nl

**Running title**: TREM1/3 in tissue repair

**Supplementary figures**

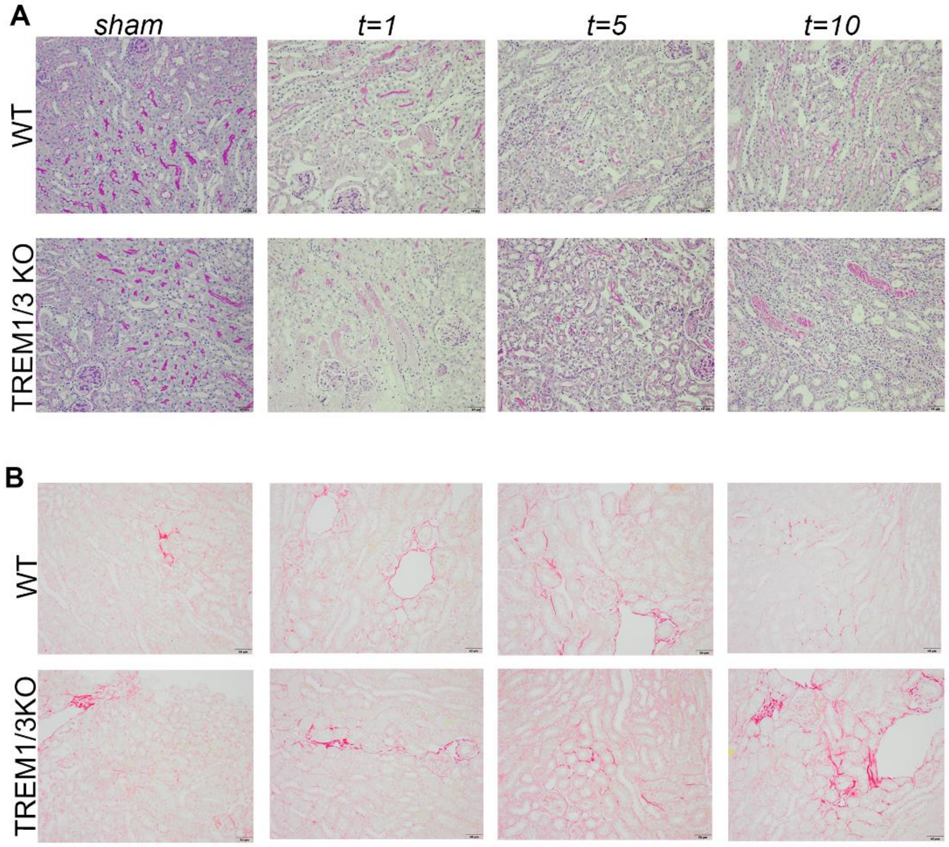


**SF1:** *Tubular damage and collagen deposits in WT and TREM1/3 KO animals***.** (A) Representative images of PAS-D staining in sham conditions and 1,5 and 10 days after mild AKI experiments. (B) Representative images of collagen deposits detected by picro sirius red hidtological staining in mild AKI experiments.

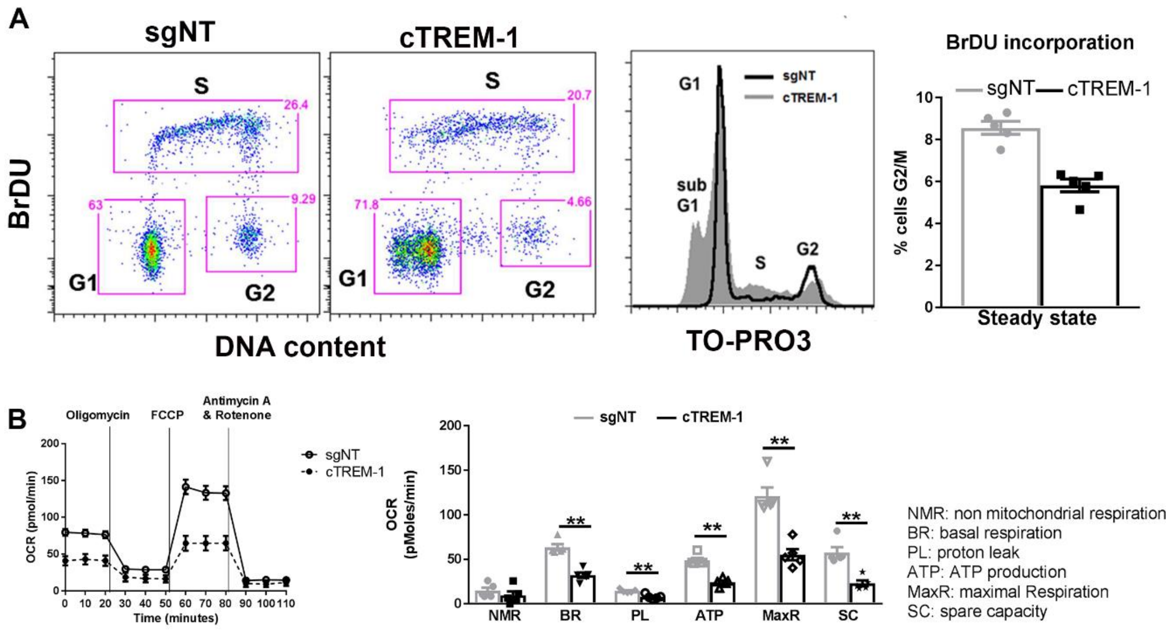


**SF2:** *TREM-1 silencing in tubular epithelial cell line (IM-TECs) results in impaired cell proliferation and mitochondrial metabolism at steady state***.** TREM-1 stable KO IM-TECs by CRISPR/Cas9 technology. (A) TREM-1-deficient TECs were cultured for 24 hours and subjected to a BrDU incorporation assay for cell cycle analysis. Histograms and polychromatic plots are shown for the 3 cell-cycle phases. Graphical visualization of the percentage of cells in G2/M. (B) Mitochondrial respiration measured with a standard protocol (Mitostress test) by Seahorse. TECs were analyzed in replicates of 5. All data are expressed as mean ± SEM and the unpaired t test was used to determine statistical differences. *P<0.05, **P<0.01

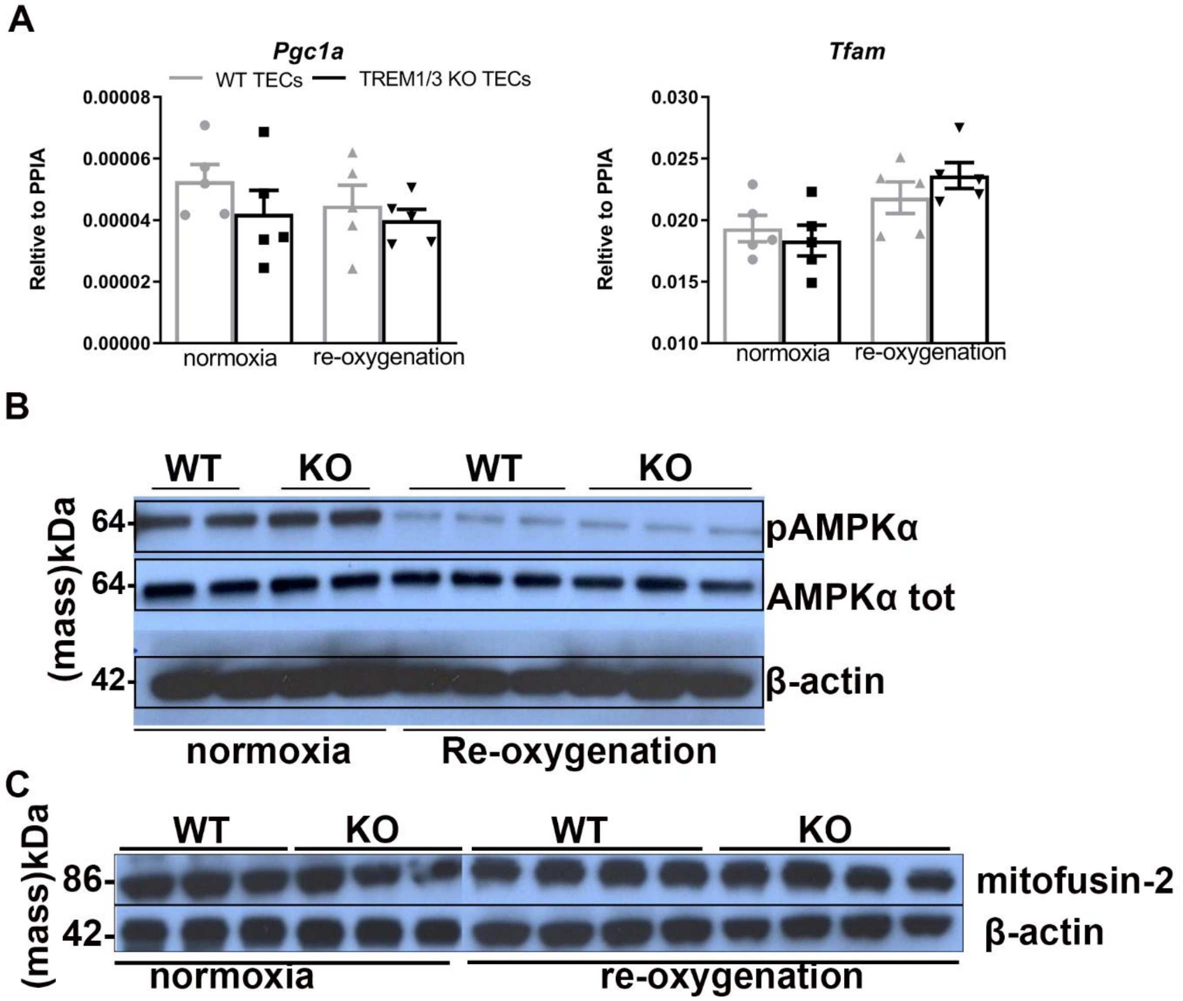


**SF3:** *TREM1/3 does not affect mitochondrial biogenesis or dynamics in primary TECs.* (A) Expression of genes involved in mitochondrial biogenesis (*Tfam* and *Pgc1a*) measured by RT-PCR. (N=5 animals per group). (B) Western blot for total and phosphorylated AMPK. (C) Western blot for Mitofusin-2 in WT and KO primary TECs under CTR conditions and after IR . All data are expressed as mean ± SEM**.**

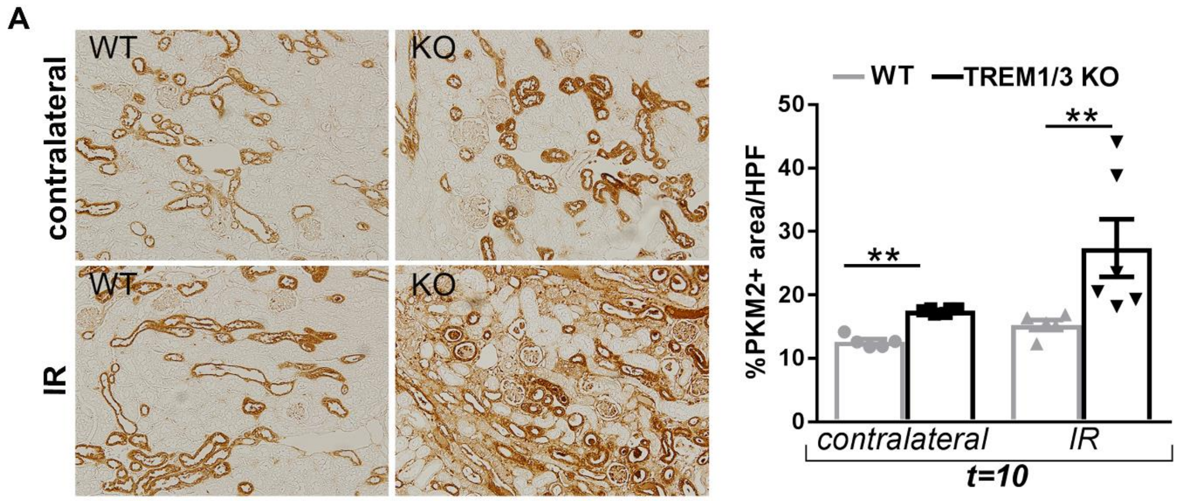


**SF4:** *Maladaptive repair in TREM1/3 KO mice correlates with mitochondrial pathology and metabolic reprogramming.* (A) Pyruvate kinase isozymes M2 detected by IHC in contralateral and ischemic kidneys from WT and TREM1/3 KO mice 10 days post-IR. Results are shown as % of positive area per HPF. All data are expressed as mean ± SEM and the Mann-Whitney test was used to determine statistical differences. **P<0.01.

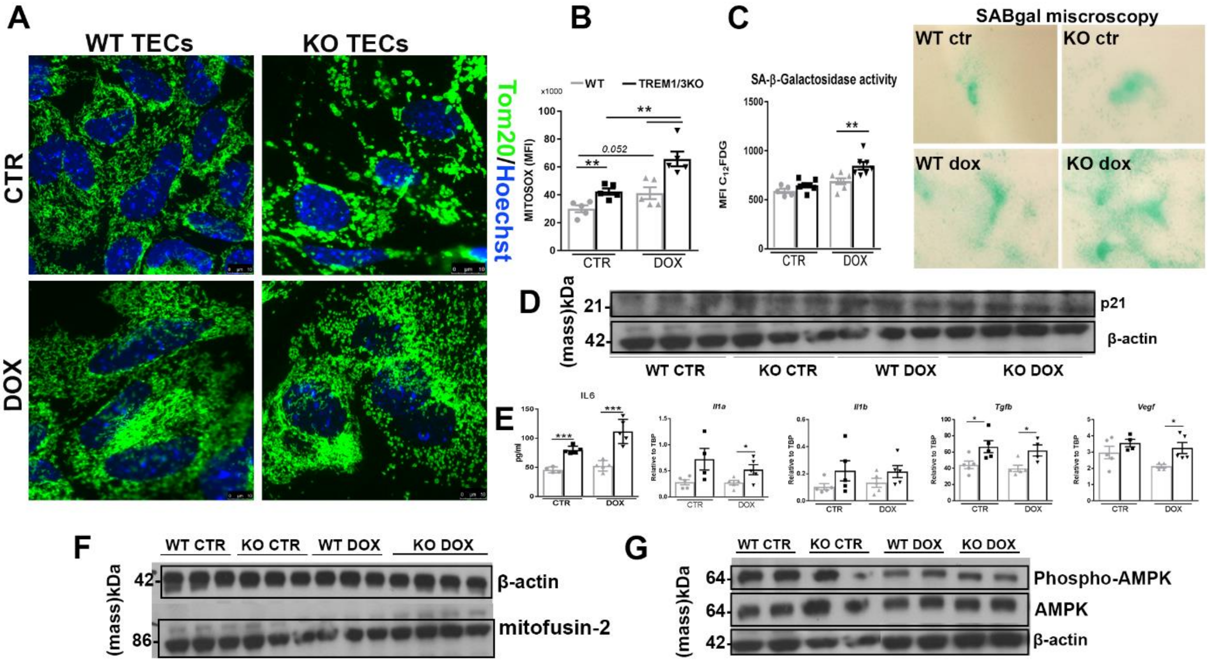


**SF5:** *Mitochondrial dysfunction and senescence in DOXO-treated TREM1/3 KO TECs.* (A) Representative immunofluorescent images of primary TECs stained for mitochondrial protein TOM20 (FITC) and Hoechst (nuclear staining), under CTR conditions and after DOXO treatment (0.1µM 24h) (magnification 40X)(N=3 animals per conditions). (B) Mitochondrial ROS production measured by FACS analysis with MitoSox probe. Results are shown as mean fluorescence intensity. (C) FACS analysis of senescence-associated β-galactosidase (SA-βgal) assay by $C_{12}$FDG incorporation in WT and TREM1/3 KO primary TECs under CTR and DOXO-treated conditions (N=7). SA-βgal staining obtained in cells cultured on coverslips. Blue/green cells represent senescent cells. (D) p21 protein expression showed by western blot in primary TEC lysates isolated from WT and TREM1/3 KO in CTR conditions and after DOXO treatment. (E) Transcript expression of SASP-components such as pro-inflammatory (Il1a, Il1b) and profibrotic genes (Tgfb, Vegf) and IL-6 release measured by ELISA**.** (F-G) Western blot for Mitofusin-2, total and phosphorylated AMPK in WT and KO primary TECs under CTR and DOXO conditions. All data are expressed as mean ± SEM and two-tailed unpaired t test was used to determine statistical differences. *P<0.05, **P<0.01.

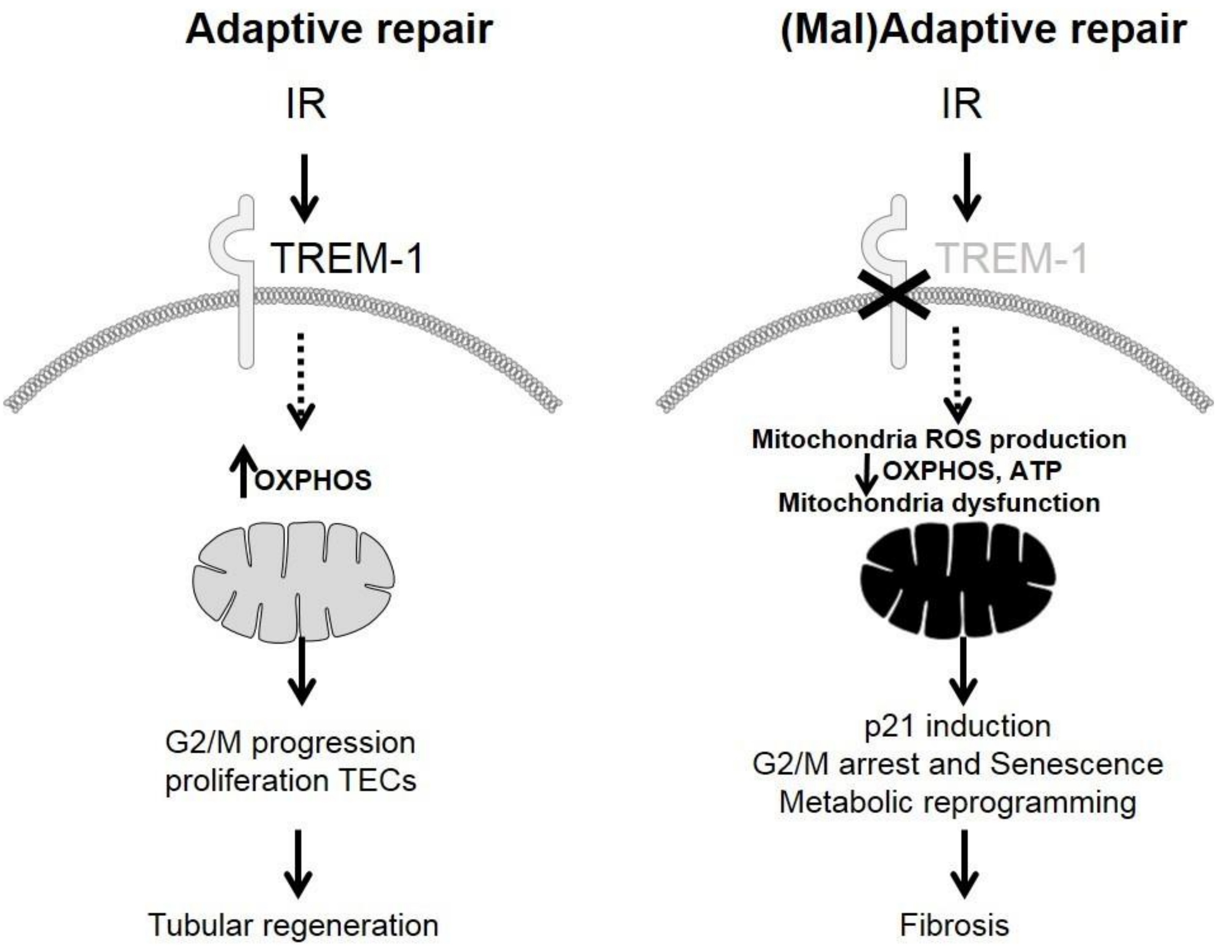


**SF6:** *Proposed mechanism of TREM1/3-mediated adaptive renal repair***.** Following IR, TREM-1 is upregulated in TECs during active repair, possibly to promote tubular regeneration by controlling mitochondrial oxidative phosphorylation (OXPHOS), which is necessary for G2/M progression. In absence of TREM1/3, TECs display an impaired mitochondrial homeostasis with elevated ROS generation and reduced usage of OXPHOS and of other metabolic pathways. This may suggest that impaired mitochondrial metabolic flexibility underlie the G2/M arrest and the onset of TEC senescence leading to maladaptive tubular repair.

## Supplementary Table 1

| **Gene** | **Primer sequences** |
|---|---|
| *Tata box binding protein (Tbp)* | F: 5’-GGAGAATCATGGACCAGAAC<br>R: 5’-GATGGGAATTCCAGGAGTCA |
| *Peptidylprolyl Isomerase A (Ppia)* | F: 5’-ATGCCAGGGTGGTGACTTTAC<br>R: 5’-GATGCCAGGACCTGTATGCT |
| *Neutrophil gelatinase–associated lipocalin (Ngal)* | F: 5’-GCCTCAAGGACGACAACATC<br>R: 5’-CTGAACCAATTGGGTCTCGC |
| *Kidney injury molecule-1 (Kim1)* | F: 5’-TGGTTGCCTTCCGTGTCTCT<br>R: 5’-TCAGCTCGGGAATGCACAA) |
| *Interleukin-1b (il1b)* | F: 5′-CTGCAGCTGGAGAGTGTGGAT<br>R: 5′-GCTTGTGCTCTGCTTGTGAG |
| *Interleukin6 (il6)* | F: 5′-GCTACCAAACTGGATATAATGGA<br>R: 5′-CCAGGTAGCTATGGTACT6CCAGAA |
| *Interleukin1a (il1a)* | F: 5’-CGCTTGAGTCGGCAAAGAAAT<br>R: 5’-TGATACTGTCACCCGGCTCT |
| *Vascular endothelial growth factor (Vegf)* | F: 5’-GCCCGGGCCTCGGTT<br>R: 5 -AACTTGATCACTTCATGGGACTTC |
| *Transforming growth factor β1 (Tgf)* | F: 5’-GCAACATGTGGAACTCTACCAGAA<br>R: 5’-GACGTCAAAAGACAGCCACTCA |
| *Connective tissue growth factor (Ctgf)* | F: 5’-TGACCTGGAGGAAAACATTAAGA<br>R: 5’-AGCCCTGTATGTCTTCACACTG |
| *Mitochondrial transcription factor A (Tfam)* | F: 5’-TCGCATCCCCTCGTCTATCA<br>R: 5’-CCACAGGGCTGCAATTTTCC |
| *Peroxisome proliferator-activated receptor gamma coactivator 1-α (Pgc1a)* | F: 5’-GAGCGAACCTTAAGTGTGGAA<br>R: 5’-TCTTGGTTGGCTTTATGAGGA |
| *Glutathione peroxidase 1 (Gpx1)* | F: 5’-TTCGGACACCAGGAGAATGG |

| | |
|---|---|
| | R: 5'-TAAAGAGCGGGTGAGCCTTC |
| *Glutathione peroxidase 3 (Gpx3* | F: 5'-CTCGGAGATACTCCCCAGTCT<br>R: 5'-TGGGAGGGCAGGAGTTCTTC |
| *Superoxide dismutase 2, mitochondrial (SOD2)* | F: 5'-ACAACTCAGGTCGCTCTTCAG<br>R: 5'-TCCAGCAACTCTCCTTTGGG |
| *cytochrome C (CytC)* | F: 5'-TCCATCAGGGTATCCTCTCC<br>R: 5'-GGAGGCAAGCATAAGACTGG |
